# Europe must open with extreme caution this summer

**DOI:** 10.1016/j.lanepe.2021.100176

**Published:** 2021-07-09

Recovery from the pandemic was a major topic of discussion at the June 2021 G7 Summit, the annual meeting of leaders of the seven prominent economies in the world: Canada, France, Germany, Japan, Italy, the UK, and the USA. Before the start of the meeting, the UK Prime Minister and host of the summit, Boris Johnson, said that the leaders had a chance “to learn lessons from the coronavirus pandemic and ensure they do not repeat the errors made during it”. Simultaneously, rising case numbers of the new SARS-CoV-2 Delta variant delayed the final lifting of restrictions in the UK. Meanwhile, eleven major European cities held their first mass gathering events since the pandemic by hosting the UEFA 2020 Football Championship with its final match on July 11, 2021. Additionally, the controversial vaccine passports allow European cross-border summer holidays in the European Union (EU) from July 1. The urge to return to normality after more than 1 year of on-and-off lockdowns is understandable but restrain is needed to prevent subsequent waves of infection. However, instead of being precautious, expectations to lift all preventive measures quickly are evident throughout Europe.

When the nationwide incidence of COVID-19 fell below a previously defined legal cut-off in Germany, school face-mask requirements were lifted in some regions, and a date to abandon the use of face masks completely became a hot topic for discussion among politicians. In neighbouring Belgium, political leaders similarly outbid each other with timelines to universally lift all restrictions, including indoor face-mask requirements, and the Netherlands plan to unmask before the end of summer. The biggest mistake countries can now make is to believe that the pandemic is over, as was observed in India, leading to a devastating second wave in May, 2021. Moving too quickly towards normalcy without caution will only delay the return to normal life—eg, in the UK so-called Freedom Day has been postponed from June 21 to July 19 due to the rapid spread of the SARS-CoV-2 Delta variant.

99% of sequenced cases in the UK are now attributable to the Delta variant, overtaking the SARS-CoV-2 Alpha variant, which had been dominant since December, 2020. Based on the speed at which the Delta variant overtook the Alpha variant, it is around 2·5 times more transmissible, with an upper estimate of up to 4 times higher transmissibility than the original SARS-CoV-2 virus. Initial analysis of Delta variant transmission in Scotland was published recently as a Correspondence in the Lancet and sheds light on the effect of the variant on hospitalisation rates and vaccine effectiveness, highlighting the need for serious caution in the rest of Europe and beyond. People infected with Delta variant are about twice as likely as those infected with Alpha to be admitted to the hospital. Vaccine effectiveness data show a reduction in preventing symptomatic disease caused by the Delta variant, particularly for the AstraZeneca vaccine. Additionally, a single vaccination dose is less protective than two vaccine doses, underscoring the importance of speeding up full vaccination coverage before rushing to normalcy. As of July, only 35% of adults in the EU and European Economic Area (including Iceland and Norway) were fully vaccinated, and with coverage barely reaching 10% in Russia and other former Soviet Union countries, a large proportion of Europe remains susceptible to infection. It is predicted that the Delta variant may account for 90% of all COVID-19 cases in the EU by the end of August and European Centre for Disease Prevention and Control (ECDC) has released a risk assessment urging countries to strictly adhere to public health measures which previously worked to control the impact of other variants.

With more than 1 year of pandemic data to review, countries have the necessary resources to implement the right strategy for defence. Two studies published in this issue of *The Lancet Regional Health – Europe*, by Ullrich and colleagues and Oh and colleagues, analysed German surveillance data of SARS-CoV-2 and other infectious diseases during 2020. Their analysis shows not only a significant decrease in the spread of SARS-CoV-2, but also a decrease in the incidence of measles, influenza and tuberculosis after implementation of non-pharmaceutical interventions (NPIs) including cancellation of mass gatherings, school closures, and a strict contact ban and closure of all non-essential businesses. These data support a positive impact of NPIs on curbing and preventing infections.

Focused preventive measures, such as mask wearing, social distancing, and avoidance of mass gatherings will prevent the mistakes made at the start of 2021, when many countries failed to contain the Alpha variant, leading to a third wave of infections. Learning this lesson will be an investment for the future because as long as the virus rages elsewhere in the world—eg, in Africa vaccination coverage is around 1%—potential for the emergence of more variants will exist and preparedness to contain them will be needed. Public officials in Europe seem reluctant to adopt a face-mask wearing culture, which is now prevalent as an effective measure to curb outbreaks in Asian countries including China and Japan where lessons have been learnt after earlier infectious disease outbreaks, notably the influenza epidemics of 1918, 1957, and 1968 and the SARS-CoV-1 epidemic in 2002. For European countries to pass the pandemic test, it will prove worthwhile to cultivate a similarly positive attitude towards face masks, such that reinstating face covering when needed can be a reflex instead of a mandate. A societal change in attitude towards pandemic preparedness in general and towards simple but effective NPIs can be a valuable tool to control future outbreaks early, including yearly influenza epidemics. This summer is a crucial time to test whether valuable lessons from the past course of the pandemic have been learnt.

*The Lancet Regional Health – Europe*

